# Hepatoprotective Effect of Oyster Peptide on Alcohol-Induced Liver Disease in Mice

**DOI:** 10.3390/ijms23158081

**Published:** 2022-07-22

**Authors:** Xueqin Wang, Huahua Yu, Ronge Xing, Pengcheng Li

**Affiliations:** 1 CAS and Shandong Province Key Laboratory of Experimental Marine Biology, Center for Ocean Mega-Science, Institute of Oceanology, Chinese Academy of Sciences, Qingdao 266071, China; xueqinwang@qdio.ac.cn (X.W.); yuhuahua@qdio.ac.cn (H.Y.); xingronge@qdio.ac.cn (R.X.); 2 Laboratory for Marine Drugs and Bioproducts, Pilot National Laboratory for Marine Science and Technology (Qingdao), No. 1 Wenhai Road, Qingdao 266237, China

**Keywords:** oyster peptide, alcohol-induced liver diseases, hepatoprotective effect, oxidative stress, inflammatory response

## Abstract

Alcohol-induced liver disease (ALD) has become one of the major global health problems, and the aim of this study was to investigate the characterization of the structure as well as the hepatoprotective effect and mechanism of oyster peptide (OP, MW < 3500 Da) on ALD in a mouse model. The results demonstrate that ethanol administration could increase the activities of aspartate aminotransferase (AST), alanine transaminase (ALT), γ-Glutamyl transferase (GGT), reactive oxygen species (ROS), malondialdehyde (MDA), and triglycerides (TG), as well as increase the interleukin-1β (IL-1β), interleukin-6 (IL-6), and tumor necrosis factor (TNF-α) levels (*p* < 0.01), and reduce the activity of superoxide dismutase (SOD) and the concentration of glutathione (GSH). Those changes were significantly reversed by the application of different doses of OP. Furthermore, the mRNA expressions of nuclear factor elythroid 2-related factor 2 (Nrf2), heme oxygenase-1 (HO-1), and quinone oxidoreductase1 (NQO1) were significantly up-regulated in OP groups, and the mRNA expressions of nuclear factor kappa-light chain enhancer of B cells (NF-κB), TNF-α, and IL-6 were markedly reduced in OP groups compared to that of the model group. Thus, OP had a significant protective effect on ALD through the enhancement of the in vivo antioxidant ability and the inhibition of the inflammatory response as possible mechanisms of action, which therefore suggests that OP might be useful as a natural source to protect the liver from alcohol damage.

## 1. Introduction

It is well known that chronic and excessive alcohol intake can lead to a number of chronic diseases, such as alcohol-induced liver disease (ALD) [1]. ALD, as one of the most prevalent types of liver disease worldwide, is a broad term that includes a range of diseases of different severities, such as steatosis, hepatitis, alcoholic cirrhosis, etc., and is present in more than 90% of heavy drinkers [2]. Among ALD patients, 25–35% develop steatohepatitis, and 8–20% eventually develop fibrosis and cirrhosis [3]. According to the Global Burden of Disease (GBD) study in 2019, alcohol contributes to the global burden of cirrhosis by 24% [4], and death from liver cancer due to alcohol represents 0.16% of all deaths. Data from 2019 were made available in October 2020 and can be accessed through this website: http://www.healthdata.org/gbd/2019 (accessed on 7 July 2022). Moreover, liver cirrhosis demonstrated marked variations between countries and regions. The alcohol-attributable proportion of liver cirrhosis was highest in India (37.12%), followed by South Korea (23.15%), and Japan (19.11%), and the lowest proportions were found in Iran and Turkey (2.48%) [3]. In addition, the cirrhosis rates increase year by year and, from 2007 to 2014, the proportion of patients with alcoholic cirrhosis with ≥3 cirrhosis complications increased from 11.6% in 2007 to 25.8% in 2014 [5]. In China, alcohol abuse has been considered the second leading cause of liver disease [6]. Therefore, it is urgent to study the treatment of ALD.

The mechanisms underlying the development of different stages of ALD are not completely understood. Some studies showed that the liver disease of a patient was likely triggered by a host of proinflammatory cytokines [7], such as interleukin-1β (IL-1β), interleukin-6 (IL-6), and tumor necrosis factor (TNF-α), that contribute to hepatocellular damage [8]. In addition, there appears to be increasing evidence that alcohol toxicity may be associated with increased oxidative stress and free radical-associated injury [9]. The oxygen metabolites are confirmed to play an important role in the pathogenesis of alcoholic liver injury, and the antioxidant system, which includes primary antioxidant enzymes such as superoxide dismutase (SOD) and glutathione peroxidase (GSH-Px), shows potential for protecting the body against those oxidants [10]. Furthermore, the lowering of glutathione (GSH) by chronic ethanol treatment has been a more consistent observation and appears to be a key lesion contributing to ALD.

It is well known that ALD is a significant burden on one’s health, while few therapeutic advances have been made in the last 40 years [11]. The main therapy for patients with all stages of ALD is alcohol cessation [12]. In addition, pharmacologic therapy including baclofen, naltrexone, disulfiram, and sodium oxybate have proven to be effective for ALD, although they have many contraindications [13]. Dimethyl diphenyl bicarboxylate has the pharmacological effects of protecting hepatocytes and increasing the detoxification function of the liver; some studies have confirmed that dimethyl diphenyl bicarboxylate has a protective effect on the liver [14]. Of course, psychotherapy is an effective method to reduce alcohol consumption and alcohol-related morbidity and mortality [15]. In addition, most patients with liver disease suffer from malnutrition due to anorexia and dysgeusia, and malnutrition is directly correlated with the degree of liver disease and mortality [16]. In addition, substantial alcohol may become the preferential fuel in hepatic cells which may turn it into an important energy source instead of into fat, which supports fatty acid accumulation [17]. It is confirmed that enteral nutrition and oral nutritional supplements could improve various aspects of malnutrition in patients with ALD [18]. Some studies showed that a long-term oral supplementation of branched chain amino acids (BCAA) could improve survival rates and reduce the frequency of major complications for patients with liver disease [19], because the BCAA could increase serum albumin concentration, which is a good nutritional parameter in hepatopaths [20].

The oyster species, *Crassostrea talienwhanensis*, is rich in protein and consists of well-balanced amino acids. In addition, the contents of functional polypeptide, glycogen, polyunsaturated fatty acid, vitamin B_2,_ and vitamin B_12_ in oyster meat are high, and the contents of trace elements needed by the human body such as zinc, iron, copper, iodine, and selenium are also relatively high [21]. In recent years, a variety of biological activities were obtained from *C. talienwhanensis* extracts, including antioxidant activity [22], anti-inflamatory activity [23], antihypertensive activity [24], and the improvement of the learning and memory ability [25]. Furthermore, oyster extract and oyster polypeptide complex have been proven to fight against alcohol-induced liver injury in mice [26]; however, the mechanisms of oyster extract on the protection of the liver and on the lowering of fat accumulation needs to be fully explored. Herein, we prepared oyster peptide in our previous work, and further studied the protective effects and mechanisms of oyster on ALD.

## 2. Results and Discussion

### 2.1. Characterization of the Structure of Oyster Peptide (OP)

#### 2.1.1. Particle Size Distribution and Zeta Potential Measurement

**Figure 1:**
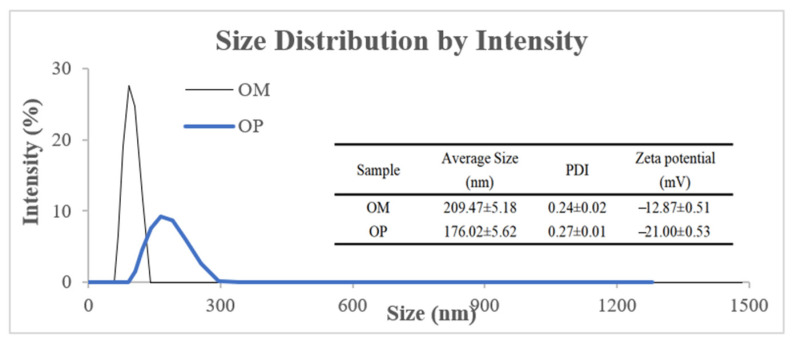
Particle size distribution and zeta potential of OM and OP.

As shown in Figure 1, the average particle size of oyster meat (OM) and OP presented a unimodal distribution, and the average particle size of OM was 209.47 nm, while by enzymatic hydrolysis, the average particle size of OP was reduced to 176.02 nm, which reflected that the particle size of OM was reduced by enzymatic hydrolysis. In addition, zeta potential is an important index used to characterize the stability of a colloidal dispersion system [27]. As shown in Figure 1, the zeta potential of OP (−21.00 mV) was larger than that of OM (−12.87 mV), which usually means that the system of OP is more stable. In addition, the polydispersity index (PDI) of OM and OP was 0.24 and 0.27, respectively, indicating that they had good dispersion.

#### 2.1.2. Fourier Transform Infrared (FTIR) Analysis

**Figure 2:**
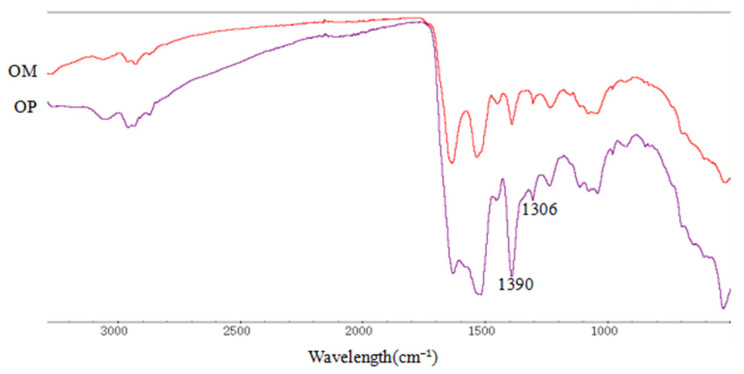
FTIR of OM and OP.

The FTIR analysis of OM and OP was performed over a range from 3000 cm^−1^ to 500 cm^−1^ to investigate the variations in the functional peaks (Figure 2). It was seen that both OM and OP showed distinctive spectra of a typical protein molecule, and the spectra of OM and OP were similar in the range from 3000 cm^−1^ to 500 cm^−1^. However, the peak heights were significantly decreased at 1390 cm^−1^ and 1306 cm^−1^, which might be due to the presence of C-H stretching vibrations [28]. In addition, the other characteristic peaks were not significantly different; only the peak areas were slightly different.

#### 2.1.3. Circular Dichroism

**Figure 3:**
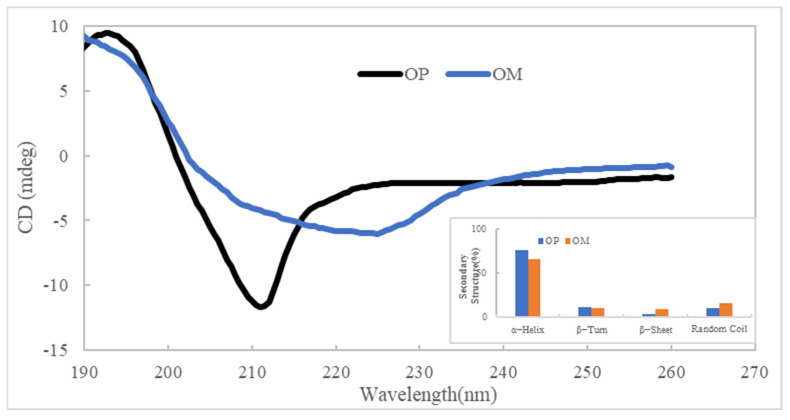
Circular dichroism of OM and OP.

Circular dichroism analysis technology is commonly used in the secondary structure of proteins, and the circular dichroism spectrum often reflects the circular dichroism of peptide bonds [29]. As shown in Figure 3, there was a positive peak around 190 nm and a negative groove from 210 nm in the circular dichroism spectrum of OP, but the circular dichroism spectrum of the OM had no distinct negative groove. According to software analysis, the samples of OM and OP both contained four secondary structures, including α-helix, β-turn, β-sheet, and random coil, and there was a slight difference in their content. Compared to the OM, the content of α-helix in the OP was increased, and the content of the random coil was decreased, which indicates that some changes in the oyster protein’s secondary structure were made through enzymatic hydrolysis.

#### 2.1.4. SEM Analysis

**Figure 4:**
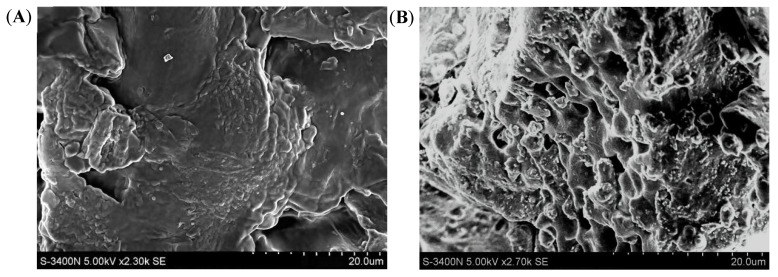
**The** SEM at 20 μm size of the OM and OP. (**A**): Sample of OM, (**B**): Sample of OP.

In order to observe the surface morphology of the OM and the OP more directly, an SEM analysis was chosen. As shown in Figure 4, compared with the OM, the surface morphology of the OP changed visibly. The OM had a relatively smooth surface and a compact structure; however, after enzymatic hydrolysis, there were many pores in the sample of the OP whose structure was loose, exposing more functional groups (Figure 4B). It can be concluded that enzymatic hydrolysis destroyed the surface structure of the OP and converted it into a small molecule polypeptide structure [29].

### 2.2. OP Groups Attenuated the Alcohol-Induced Liver Injury

**Figure 5:**
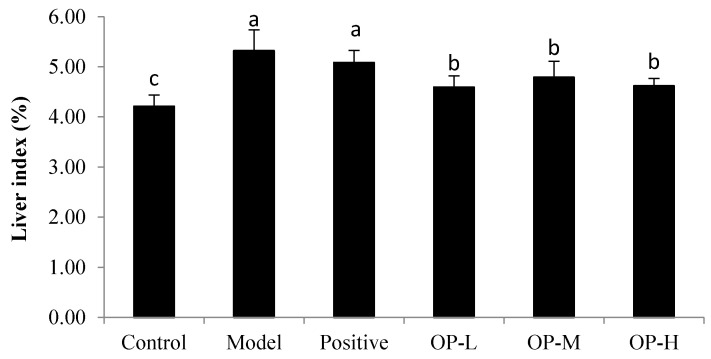
Effects of OP on the liver index of ALD mice (means ± SD, *n* = 10). Bars with different superscripts (a, b, and c) were significantly different (*p* < 0.05) according to variance analysis (ANOVA) using the SPSS software (IBM SPSS Statistics 22, IBMCorp., Armonk, NY, USA) and means were compared by Duncan’s multiple comparison post-test.

Liver index was the mass ratio calculated by the liver weight to the body weight. As shown in Figure 5, compared with that of the control group, the model group showed a significant increase in the weight of the mice livers (*p* < 0.05) of 26.29%, which meant that continuous ethanol gavage caused liver enlargement in mice. The positive group could not significantly improve the liver index of mice compared to the model group. Although liver indices in the different OP groups were always higher than in the control group, they significantly decreased by 13.67%, 9.97%, and 13.20%, respectively, compared to the model group (*p* < 0.05). It was confirmed that ethanol could influence the weight of mice livers, but the OP would relieve liver enlargement in ALD mice.

**Figure 6:**
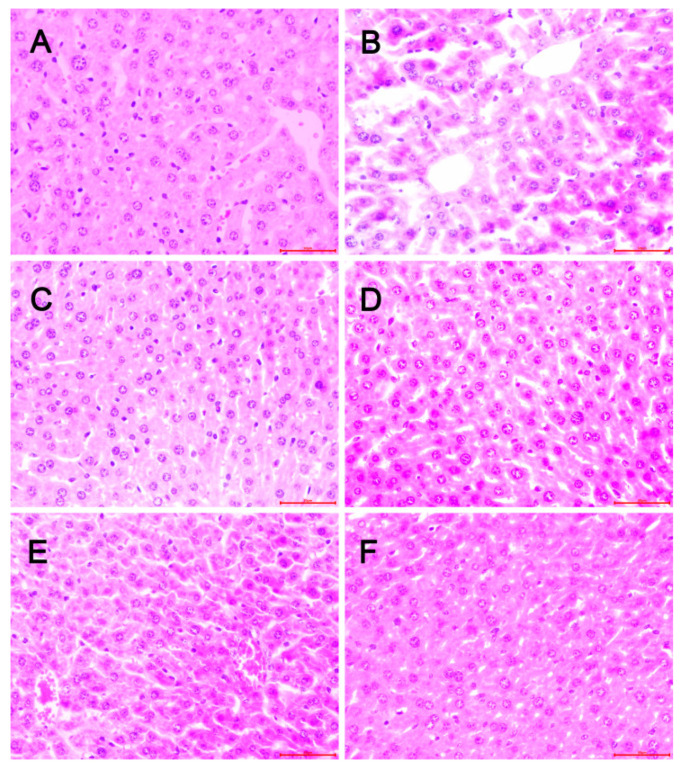
Effect of OP on liver histopathological changes in ALD mice. (Original magnification ×40). (**A**): Control group; (**B**): Model group (10 mL/kg bw ethanol); (**C**): Positive group (10 mg/kg bw dimethyl diphenyl bicarboxylate + 10 mL/kg bw ethanol); (**D**): OP-L group (120 mg/kg bw OP + 10 mL/kg bw ethanol); (**E**): OP-M group (240 mg/kg bw OP + 10 mL/kg bw ethanol); (**F**): OP-H group (480 mg/kg bw OP + 10 mL/kg bw ethanol).

In order to confirm the protective effect of OP on hepatic steatosis induced by chronic ethanol intake, liver histological analysis was observed using H&E staining. As shown in Figure 6, there were no pathologic changes seen in the control group (Figure 6A); however, lobular inflammation, fatty accumulation, hepatocellular swelling, and the loss of cellular boundaries were observed in the model group (Figure 6B), which showed a typical pattern of massive large droplets of fat, suggesting that ethanol exposure for 6 weeks might cause a decrease in very low-density lipoprotein (VLDL) synthesis to account for the greater amount of lipids in the hepatocyte [30]. In contrast, these pathologic changes were ameliorated in the OP groups; the OP-H group showed especially well-preserved cytoplasm and legible nucleoli (Figure 6F), possibly due to the impairment of VLDL synthesis in the OP-H group, resulting in a lower rate of triglyceride (TG) transport from the liver. However, the liver histopathology showed scattered droplets of fat in the positive group and in the OP-L group (Figure 6C,D). Overall, from the pathology results, we can conclude that different doses of OP groups may protect the liver structure in the ALD mice, as well as inhibit the synthesis of lipids in the liver [31].

### 2.3. Effects of OP on Serum Aminotransferase Activities in Mice

**Figure 7:**
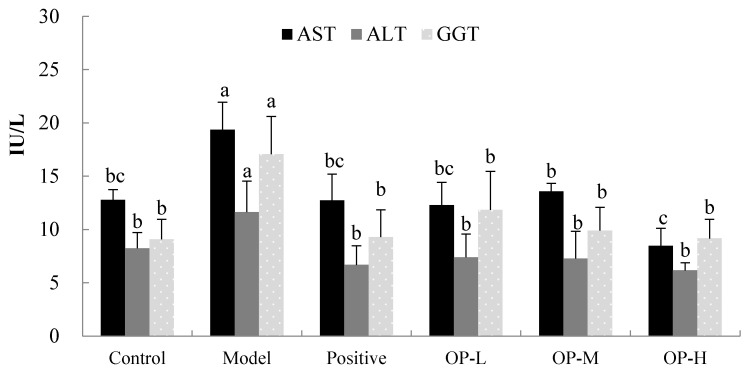
Effect of OP on the activities of AST, ALT, and GGT (means ± SD, *n* = 10). Bars with different superscripts (a, b, and c) were significantly different (*p* < 0.01) according to ANOVA using the SPSS software and means were compared by Duncan’s multiple comparison post-test.

The activities of aspartate aminotransferase (AST) and alanine transaminase (ALT) are considered as reliable and sensitive indicators of ALD [32]. When the structural integrity of the hepatic cells are damaged, soluble enzymes such as ALT and AST are released into the blood and show high levels of activity [33]. The results of the hepatoprotective effect of the OP on the serum AST and ALT activities are shown in Figure 7. In the control group, the serum AST and ALT activities were 12.78 ± 0.96 IU/L and 8.24 ± 1.47 IU/L, respectively, and in the model group, the serum AST and ALT activities were 19.35 ± 2.59 IU/L and 11.64 ± 2.89 IU/L, respectively, which were significantly increased by 51.42% and 41.26%, respectively, as compared to that of the control group (*p* < 0.01), indicating that continuous ethanol gavage caused liver injury. After the administration of different doses of OP for 6 weeks, the activities of AST and ALT were significantly decreased in the OP groups as compared to the model group (*p* < 0.01). In the OP-H group especially, the AST and ALT activities were significantly reduced by 56.26% and 47.04%, respectively, as compared to the model group (*p* < 0.01).

In addition, γ-Glutamyl transferase (GGT) has also been regarded as a biological marker of heavy ethanol consumption or hepatobiliary disease such as fatty liver [34]. As shown in Figure 7, ethanol administration significantly increased the activity of GGT in the model group by 88.11%, compared to that of the control group (*p* < 0.01). Kim et al. [35] found that alcohol causes hepatic damage via up-regulated GGT linked with the reduced GSH in the unbalanced redox system of the liver, and the redox system also includes other antioxidant enzymes, such as SOD, GSH-Px, glutathione reductase (GR), and glutathione-S-transferase (GST) [36]. The administration of dimethyl diphenyl bicarboxylate and different doses of OP along with ethanol significantly reversed the GGT level near to that of the control. In the OP-H group especially, the GGT activity was significantly reduced by 46.34% as compared to the model group (*p* < 0.01). In conclusion, this result shows that the daily administration of OP improved hepatic tissue damage caused by ethanol in mice.

### 2.4. Effects of OP on TG

**Figure 8:**
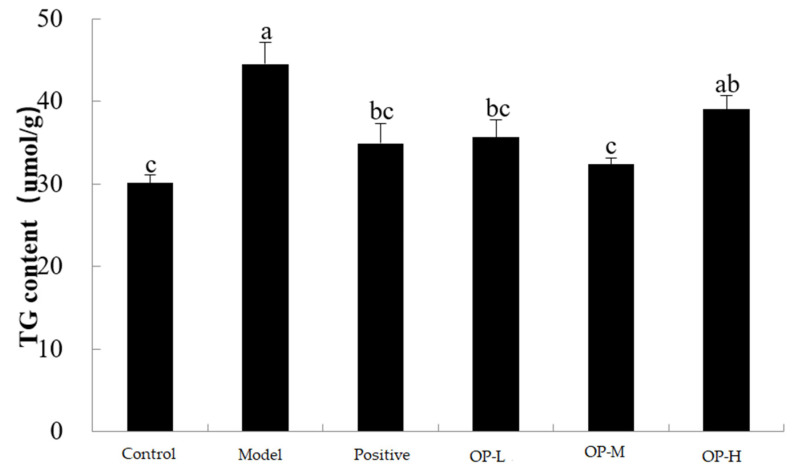
Effect of OP on TG content (means ± SD, *n* = 10). Bars with different superscripts (a, b, and c) were significantly different (*p* < 0.01) according to ANOVA using the SPSS software and means were compared by Duncan’s multiple comparison post-test.

TG is the liver damage indicator usually used to evaluate the degree of hepatocyte degeneration. The TG content is presented in Figure 8. In comparison with the control group, the TG levels in the model group were significantly elevated by 47.90% (*p* < 0.05), which indicated that ethanol exposure might promote the accumulation of triglycerides. Moreover, after the treatment with OP, the TG contents in different doses of OP groups were visibly decreased in comparison with the model group, and the TG content in the OP-M group was significantly decreased by 27.35%, especially (*p* < 0.01) while there was no dose-dependent relationship with the TG contents in the OP groups. The results demonstrate that OP could dramatically decrease lipid accumulation and reduce the degree of hepatocellular steatosis in hepatic tissues in alcohol-induced fatty liver in mice.

### 2.5. Effects of OP on Alcohol-Induced Oxidative Stress in Mice

Several studies have investigated that long-term excessive alcohol can cause oxidative stress in the liver [37]. When the mice were force-fed alcohol for a long time, the reactive oxygen species (ROS) production was enhanced in the mice liver tissue. Because the ethanol impaired the mitochondrial structure and function, it resulted in an increase in ROS production [38]. However, when ROS generation grows beyond the capacity of the cellular antioxidant system, it results in protein and lipid oxidation and ultimately leads to cell death [39]. Nevertheless, the redundant ROS could be eliminated by the antioxidant enzymes, such as SOD, which play important roles in alcohol-induced liver oxidative stress [40]. Furthermore, GSH is a key intracellular antioxidant and plays a crucial role in metabolizing a large number of toxic agents, including alcohol. Alcohol-induced liver injury was associated with the decreation of hepatic actioxidant defense, especially GSH [38]. The levels of oxidative stress factors of the ALD mice are shown in Table 1. In comparison with the control group, the ROS, MDA, and TG levels in the model group were significantly elevated by 64.32%, 77.02%, and 47.90%, respectively. Conversely, the activity of SOD and the concentration of GSH were markedly decreased by 43.13% and 37.48%, respectively (*p* < 0.01).

After the administration of different doses of OP for 6 weeks, the levels of oxidative stress factors were improved and the OP groups could significantly reduce the levels of ROS and MDA, as well as increase the levels of SOD and GSH (*p* < 0.01). In comparison with the model group, the ROS and malondialdehyde (MDA) levels in the OP-H group were decreased by 36.37% and 53.45%, respectively. Conversely, the activity of SOD in the OP-H group was increased by 40.12% and the concentration of GSH in the OP-L group was increased by 44.88%. In our previous study, we confirmed that the oyster protein hydrolysates had better free radical scavenging activities, including 1,1-diphenyl-2-picryhydrazyl (DPPH) radical, hydroxyl radical, and superoxide-radical scavenging [25]. Zhang et al. [41] observed that the oyster meat hydrolysates by alcalase exhibited better antioxidant activities in vitro and in vivo. Then, we inferred that the OP could scavenge radicals and relieve oxidative stress in ALD mice, the excess ROS were eliminated, and lipid peroxidation was improved, resulting in the lipid peroxidation product MDA decreasing in the OP groups. On the other hand, with the elimination of free radicals in ALD mice, the hepatic antioxidant status was enhanced and the activity of SOD and concentration of GSH were increased.

In addition, we further studied the antioxidant stress signaling pathway involved in ALD. Oxidative stress is the common pathogenesis of many liver diseases. Nuclear factor elythroid 2-related factor 2- antioxidant response element (Nrf2-ARE) is an important signal pathway of antioxidant stress in vivo and has recently been considered as a new target for the treatment of ALD [42]. The nuclear factor elythroid 2-related factor 2 (Nrf2) could positively regulate the expression of cytoprotective genes associated with antioxidants, including heme oxygenase-1 (HO-1) and NAD(*p*)H: quinone oxidoreductase1 (NQO1) [43]. Some research found that Nrf2-null mice could reduce the constitutive expression and activity of NQO1 in the liver [44], and with the Nrf2 overexpression, the HO-1 was up-regulated in mice liver tissue [45].

**Figure 9:**
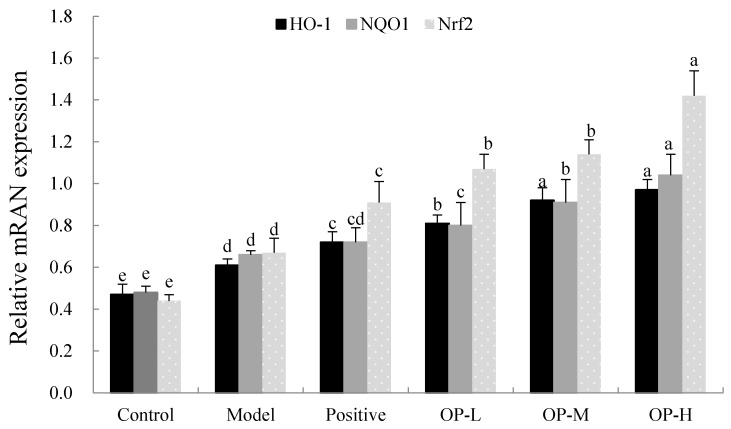
The mRNA expressions of HO-1, NQO1, and Nrf2 in liver of different groups (means ± SD, *n* = 10). Bars with different superscripts (a, b, c, d and e) were significantly different (*p* < 0.01) according to ANOVA using the SPSS software and means were compared by Duncan’s multiple comparison post-test.

As shown in Figure 9, after six weeks of ethanol administration, the mRNA expression of Nfr2, HO-1, and NQO1 was significantly up-regulated in the model group by 52.27%, 29.79%, and 37050%, respectively, compared to that of the control group (*p* < 0.05). Nrf2 bounds to redox-sensitive Keap1, and the release of Nrf2 depends on the actual oxidative state of the cell. When excess ethanol results in oxidative stress, Nrf2 dissociates from Keap1 and translocates to the nucleus to bind to ARE, and the Nfr2, HO-1, and NQO1 mRNA are commonly up-regulated following oxidative stress and cellular injury [46]. Some scholars also speculated that this is an adaptive response of the body to oxidative stress [47]. In addition, compared to the model group, the mRNA expression of Nfr2, HO-1, and NQO1 were even higher after different doses of OP treatment (*p* < 0.05). The mRNA expressions in the OP-H group were especially up-regulated by 111.94%, 59.02%, and 57.58%, respectively, compared to that of the model group, which means that the OP induced more Nrf2 for nuclear translocation, up-regulated the downstream target gene HO-1 and NQO1 expression, scavenged the oxygen free radical induced by ethanol, and improved the antioxidant capacity of liver cells.

### 2.6. Effects of OP on Alcohol-Induced Inflammatory Response in Mice

In addition to alcohol-induced oxidative stress, hepatic inflammation is another critical etiological factor with respect to liver injury caused by alcohol [48]. The enhanced inflammatory response would result in the over-production of pro-inflammatory cytokines, including IL-1β, IL-6, and TNF-α [49]. Then, we further determined the levels of pro-inflammatory cytokines IL-1β, IL-6, and TNF-α in the liver tissues of the ALD mice. As shown in Table 2, compared to the control group, a significant induction of IL-1β was observed in the model group (*p* < 0.05), and the levels of IL-6 and TNF-α were higher in the model group, which were increased by 65.33%, 27.31%, and 25.37%, respectively, compared to that of the control group. However, the different doses of the OP groups could markedly reduce IL-1β, TNF-α, and IL-6 production, and the OP-H group could significantly reduce the IL-1β and IL-6 levels by 50.29% and 29.73%, respectively. Furthermore, the OP-L group could significantly reduce the TNF-α level by 46.89%, compared to that of the model group (*p* < 0.05). These results indicate that the OP could inhibit inflammatory response in alcoholic liver injury.

Next, we investigated the molecular mechanism underlying the protective effect of OP administration against the inhibition of the production of inflammatory cytokines. The nuclear factor kappa-light chain enhancer of B cells (NF-κB) was an important transcription factor for many molecules that, in the early stages of the immune response and in all stages of inflammation, were regulated by NF-κB. When NF-κB was activated, it entered the nucleus and bound to various inflammatory factor promoters or enhancers, initiating the transcription of inflammatory mediators and participating in the inflammatory response [50]. The activation of NF-κB would lead to an increase in the expression of proinflammatory cytokines and chemokines; moreover, in alcoholic liver disease, the degree of increased NF-κB activity in the liver was closely related to the degree of inflammation in the liver [51].

**Figure 10:**
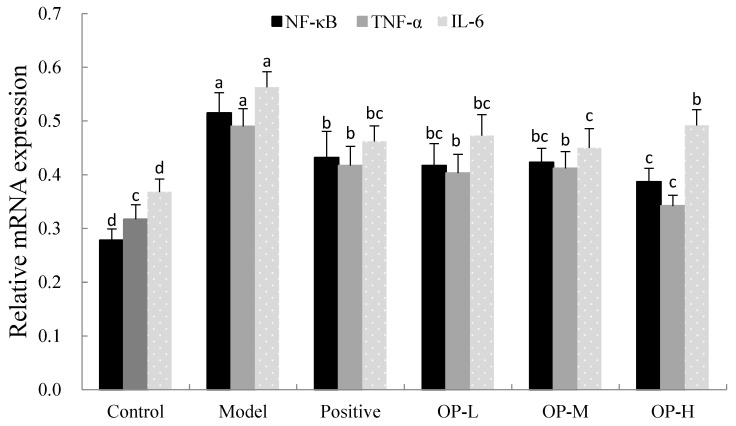
The mRNA expressions of NF-κB, TNF-α, and IL-6 in liver of different groups (means ± SD, *n* = 10). Bars with different superscripts (a, b, c and d) were significantly different (*p* < 0.01) according to ANOVA using the SPSS software and means were compared by Duncan’s multiple comparison post-test.

As shown in Figure 10, in the control group, the mRNA expressions of NF-κB, TNF-α, and IL-6 in liver tissue were low, while in the model group, the expressions of NF-κB, TNF-α, and IL-6 were significantly enhanced by 85.25%, 54.57%, and 52.99%, respectively, which suggests that the expression of NF-κB might be involved in the pathogenesis of alcoholic liver disease. Compared with the model group, the mRNA expressions of NF-κB, TNF-α, and IL-6 in the different doses of OP groups were markedly decrease, and in particular, the expressions in the OP-H group were down-regulated by 24.85%, 30.20%, and 12.61%, respectively, which means that the OP groups could inhibit the expressions of inflammatory factors and reduce the expression of NF-κB, as well as alleviate the inflammatory damage of the liver.

## 3. Material and Methods

### 3.1. Materials for Animal Experiment

Oyster (*Crassostrea talienwhanensis*) was purchased from a seafood market in Qingdao, China. Upon arrival, the oyster was washed, and the OM was separated from the shells, homogenized, and mixed with deionized water at a ratio of 1:4.8 *v*/*w*. The pH of the mixture was adjusted to 8.3 and the hydrolysis temperature was 41.7 °C; then, the trypsin was added with the enzyme concentration of 1323.8 U/g. After 6.7 h hydrolysis, the hydrolysates were centrifuged and the supernatant was separated by ultrafiltration membranes with molecular weight cut-offs of 3500 Da. Finally, the hydrolysates with molecular weight below 3500 Da were spray-dried to obtain the OP, which contained 13 peptides with high-ranking scores that were rich in Lys, Arg, His, and Thr (Table 3). The above work has been completed in our previous experiment [22].

### 3.2. Chemicals and Reagents

The ethanol (99.5% purity) was purchased from Shanghai Aladdin Bio-Chem Technology Co., LTD (Shanghai, China). Assay kits for MDA, TG, AST, ALT, GGT, GSH, and SOD, and enzyme-linked immunosorbent assay (ELISA) kits for IL-1β, IL-6, and TNF-α were purchased from Nanjing Jiancheng Bioengineering Institute (Nanjing, China). ELISA kit for hepatic tissue of ROS was purchased from Shanghai Saint-Bio Biotechnology CO., LTD (Shanghai, China). Assay kits for RNA extraction, RNA reverse transcription, and RT-PCR reaction were purchased from Baori Doctor Technology (Beijing) Co., LTD (Beijing, China).

### 3.3. Determination of Particle Size, Polydispersity Index and Zeta Potential

OM and OP solutions were prepared in quantities of 5.0 mg/mL, and the solvent was distilled water. Then, the samples (1.0 mL) were taken out of the polystyrene cell, and the particle size, zeta potential, and PDI of the samples were detected using a nanoparticle size potential analyzer (Zetasizer Nano-ZS 90, Malvern Instruments Co., Ltd., Malvern, UK). All measurements were performed at 25 °C and repeated three times.

### 3.4. FTIR Analysis

The OM and OP were investigated between 4000 cm^−1^ and 400 cm^−1^ by the spectrometer of Thermo Scientific Nicolet iS10. FTIR spectra were measured by a Nicolet Magna-Avatar 360 with KBr disks.

### 3.5. Circular Dichroism

The OM and OP (1.0 mg/mL) were subjected to circular dichroism analysis under continuous nitrogen flow conditions with a fixed wavelength between 180 and 260 nm.

### 3.6. Scanning Electron Microscope (SEM) Analysis

The SEM images were obtained in a S4800 field emission SEM (MX 2000, Cam Scan, Cambridge, England). The OM and OP were gold sputter-coated at 2.0 mA for 1 min. An accelerating voltage of 5 kV was used for all SEM observations.

### 3.7. Animal and Treatment

Ethics approval of the study was received from the Institutional Animal Care and Use Committee (IACUC No. AUP-QYCT2019016). In essence, the IACUC was responsible for ensuring that research animals at the committee’s institution were used in a humane way in procedures that were likely to improve animal or human health, advance knowledge, and be for the good of society. Ninety C57BL/6 mice (18–22 g, Approval No. SCXK 20140007) were provided by the Jinan Pengyue Experimental Animal Breeding co. LTD (Jinan, China). Four-week-old mice were allowed to adapt to their surroundings for 4 days before starting the experiments and were housed in stainless steel cages at room temperature (20–26 °C), with humidity ranging from 40–60%, and a 12/12 h light–dark cycle. The mice had free access to water and a standard rodent diet. After four days, mice were randomly assigned to the following six groups, each containing fifteen animals. Control group was orally administered a daily dose of physiological saline (10 mL/kg bw). Model group was orally administered a daily dose of 50% (*v*/*v*) ethanol (10 mL/kg bw). Positive group (PC) was firstly administered dimethyl diphenyl bicarboxylate (Beijing Union Pharmaceutical Factory, Beijing, China) in a dose of 10 mg/kg bw. After one hour, the mice received 50% (*v*/*v*) ethanol (10 mL/kg bw). Three OP groups (OP-L, OP-M, and OP-H) were firstly administered different doses of OP (120, 240, and 480 mg/kg bw, respectively). After one hour, the mice received 50% (*v*/*v*) ethanol (10 mL/kg bw). The oral administration lasted for 6 weeks.

### 3.8. Serum Analysis

During the animal experiment, some mice died due to ethanol administration and individual differences, and we chose 10 mice in each group as the number of samples for the following test. After the last gavage, the mice were sacrificed 16 h later, and blood and liver samples were collected immediately. Serum was maintained at 4 °C for 1 h and centrifuged at 1000 rpm for 15 min at 4 °C and then stored at −80 °C. Levels of ALT, AST, and GGT were determined using commercially available assay kits from the Nanjing Jiancheng Bioengineering Institute (Nanjing, China).

### 3.9. Liver Tissue Analysis

Mouse livers were weighted and dissected into two parts: one part was preserved in 4% paraformaldehyde for histological analysis, and the other liver tissue was placed in 0.9% physiological saline. The amount of physiological saline was nine times the weight of liver tissue and homogenized by tissue homogenizer, centrifuged at 3000 rpm for 15 min at 4 °C, and the supernatant was obtained for further assays. Levels of ROS, MDA, TG, SOD, GSH, IL-1β, IL-6, and TNF-α were determined using assay kits according to the manufacturer’s instructions, which were purchased from Nanjing Jiancheng Bioengineering Institute (Nanjing, China) and Shanghai Saint-Bio Biotechnology CO., LTD (Shanghai, China).

### 3.10. Liver Histological Analysis

The left middle lobes of the mice livers were embedded in paraffin, sectioned into 5 μm-thick slices, and stained using the hematoxylin and eosin (H&E) method. The sections were then analyzed under light microscopy (Olympus, Tokyo, Japan).

### 3.11. RNA Extraction and Real-Time PCR (RT-PCR)

Total RNAs were extracted from liver tissues, and total RNA extraction, reverse transcriptional reaction, and RT-PCR reaction were performed using the kit purchased from Takara Biomedical Technology Co., LTD (Beijing, China). Sequences of primers are shown in Table 4. The PCR conditions for Nrf2, HO-1, and NQO1 and β-actin were 45 cycles of 95 °C for 10 min, 95 °C for 15 s, 65 °C for 45 s. The PCR conditions for NF-κB were 39 cycles of 95 °C for 30 s, 95 °C for 10 s, 60 °C for 30 s, 72 °C for 30 s, and 65 °C for 5 s; and for IL-6 and TNF-α were 40 cycles of 94 °C for 5 min, 94 °C for 20 s, 57 °C for 20 s, 72 °C for 20 s, and 40 cycles of 94 °C for 5 min, 94 °C for 20 s, 60 °C for 20 s, 72 °C for 20 s, respectively. The results were analyzed using the 2^−ΔΔCt^ method of analysis [52].

### 3.12. Statistical Analysis

Data are presented as means ± SD. The statistical significance of the data was determined by analysis of variance (ANOVA) using the SPSS software (version 18.0 for Windows, SPSS Inc., Chicago, IL, USA) and means were compared by Duncan’s multiple comparison post-test. Statistical differences were considered to be significant at *p* < 0.05.

## 4. Conclusions

Herein, we obtained the OP (MW < 3500 Da) by enzymatic hydrolysis, and it had good particle size distribution and peptide bond characteristics. In addition, we evaluated the hepatoprotective effect of the OP on alcohol-induced liver diseases in mice and found that OP could significantly reduce the levels of AST, ALT, GGT, ROS, MDA, and TG, as well as increase the activity of SOD and the concentration of GSH compared to the model group. The results further suggest that OP had a significant protective effect on ALD by fighting against the oxidative stress and inflammatory response. This study presented persuasive results to support the hepatoprotective activity of OP.

## Figures and Tables

**Table 1:** Effect of OP on oxidative stress factors in tissues of the ALD mice.

Groups	ROS (U/mL)	SOD (U/mgprot)	GSH (nmol/g prot)	MDA (nmol/mg)
Control	49.96 ± 3.04 ^c^	136.16 ± 11.75 ^a^	550.75 ± 39.00 ^a^	0.631 ± 0.148 ^b^
Model	82.10 ± 4.18 ^a^	77.43 ± 7.93 ^c^	344.32 ± 21.61 ^b^	1.117 ± 0.250 ^a^
Positive	63.48 ± 6.97 ^b^	98.69 ± 11.62 ^bc^	416.48 ± 39.34 ^ab^	0.749 ± 0.166 ^b^
OP-L	55.88 ± 6.81 ^bc^	84.73 ± 10.95 ^bc^	498.84 ± 47.62 ^a^	0.759 ± 0.271 ^b^
OP-M	57.63 ± 8.74 ^bc^	93.41 ± 12.47 ^bc^	463.19 ± 16.37 ^ab^	0.684 ± 0.072 ^b^
OP-H	52.24 ± 3.20 ^c^	108.50 ± 11.17 ^ab^	416.84 ± 31.93 ^ab^	0.520 ± 0.098 ^b^

Values are expressed as means ± SD, *n* = 10. Values with different superscripts (a, b, and c) were significantly different (*p* < 0.01) according to ANOVA using the SPSS software and means were compared by Duncan’s multiple comparison post-test.

**Table 2:** Effect of OP on pro-inflammatory cytokines in tissues of the ALD mice.

Groups	IL-1β (ng/L)	IL-6 (ng/L)	TNF-α (ng/L)
Control	22.50 ± 3.79 ^b^	7.56 ± 1.40 ^ab^	194.40 ± 25.27 ^ab^
Model	37.19 ± 4.00 ^a^	9.62 ± 1.14 ^a^	243.72 ± 13.66 ^a^
Positive	31.80 ± 3.07 ^ab^	6.79 ± 1.23 ^b^	160.93 ± 17.57 ^b^
OP-L	20.58 ± 1.94 ^b^	8.13 ± 1.29 ^ab^	129.45 ± 11.60 ^b^
OP-M	19.57 ± 3.11 ^b^	8.52 ± 1.34 ^ab^	136.86 ± 11.31 ^b^
OP-H	18.49 ± 1.79 ^b^	6.76 ± 1.92 ^b^	141.00 ± 13.08 ^b^

Values are expressed as means ± SD, *n* = 10. Values with different superscripts (a and b) were significantly different (*p* < 0.05) according to ANOVA using the SPSS software and means were compared by Duncan’s multiple comparison post-test.

**Table 3:** The peptide sequences of OP.

No.	Sequence	Mass (Da)	Length	Parental Protein	Position
1	LAGELHQEQENYK	1557.74	13	K1QTC1	745–757
2	AIDTIINQK	1014.57	9	K1R6Z7	223–231
3	DSYVGDEAQSK	1197.52	11	Q8TA69; C4NY62	52–62
4	PGTTEDEPVK	1071.51	10	K1Q5P0	498–507
5	ETVIDTIQK	1045.57	9	K1RHA0	148–156
6	DLESQLK	831.43	7	K1QRU8	989–995
7	NAETELGETSQR	1333.61	12	K1QTC1	681–692
8	EYDESGPSIVHR	1387.64	12	Q8TA69; C4NY62	362–373
9	DSDLEGHPTPR	1222.56	11	K1RBC9	173–183
10	HDNPGDLGDLH	1188.52	11	K1PY89; K1QLW5	128–138
11	AQCEMEPNH	1114.42	9	K1PY89; K1QLW5	47–55
12	ESAGIHETT	943.42	9	Q8TA69	271–279
13	NTVLSGGTT	848.42	9	Q8TA69	297–305

**Table 4:** Sequences of primers in RT-PCR.

Primer	Sequence (5′–3′)	Lengths of Primers (bp)	Lengths of Products (bp)
Nrf2-F	CAGTGCTCCTATGCGTGAA	19	109
Nrf2-R	GCGGCTTGAATGTTTGTC	18	
HO-1-F	ACAGATGGCGTCACTTCG	18	128
HO-1-R	TGAGGACCCACTGGAGGA	18	
NQO1-F	CTTTAGGGTCGTCTTGGC	18	102
NQO1-R	CAATCAGGGCTCTTCTCG	18	
NF-kB1-F	AGCTTATGCCGAACTTCTCG	20	176
NF-kB1-R	GACTCCGGGATGGAATGTAA	20	
TNF-α-F	ACTGGCGTGTTCATCCGTTCT	21	215
TNF-α-R	CGCAATCCAGGCCACTACTTC	21	
IL-6-F	AGTTGTGCAATGGCAATTCTG	21	223
IL-6-R	AGGACTCTGGCTTTGTCTTTC	21	
β-Actin-F	CTGTCCCTGTATGCCTCTG	19	218
β-Actin-R	ATGTCACGCACGATTTCC	18	

## Data Availability

Not applicable.
